# Studies on Multifunctional Effect of All-Trans Retinoic Acid (ATRA) on Matrix Metalloproteinase-2 (MMP-2) and Its Regulatory Molecules in Human Breast Cancer Cells (MCF-7)

**DOI:** 10.1155/2009/627840

**Published:** 2009-07-19

**Authors:** Anindita Dutta, Triparna Sen, Aniruddha Banerji, Shamik Das, Amitava Chatterjee

**Affiliations:** Department of Receptor Biology & Tumor Metastasis, Chittaranjan National Cancer Institute, Kolkata 700 026, India

## Abstract

*Background*. Vitamin A derivative all-trans retinoic acid (ATRA) is considered as a potent chemotherapeutic drug for its capability of regulating cell growth and differentiation. We studied the effect of ATRA on MMP-2 in MCF-7, human breast cancer cells, and the probable signaling pathways which are affected by ATRA on regulating pro-MMP-2 activity and expression. *Methods*. Gelatin zymography, RT-PCR, ELISA, Western blot, Immunoprecipitation, and Cell adhesion assay are used. *Results*. Gelatin zymography showed that ATRA caused a dose-dependent inhibition of pro-MMP-2 activity. ATRA treatment downregulates the expression of MT1-MMP, EMMPRIN, FAK, NF-kB, and p-ERK. However, expression of E-cadherin, RAR, and CRABP increased upon ATRA treatment. Binding of cells to extra cellular matrix (ECM) protein fibronectin reduced significantly after ATRA treatment. *Conclusions*. The experimental findings clearly showed the inhibition of MMP-2 activity upon ATRA treatment. This inhibitory effect of ATRA on MMP-2 activity in human breast cancer cells (MCF-7) may result due to its inhibitory effect on MT1-MMP, EMMPRIN, and upregulation of TIMP-2. This study is focused on the effect of ATRA on MMP, MMP-integrin-E-cadherin interrelationship, and also the effect of the drug on different signaling molecules which may involve in the progression of malignant tumor development.

## 1. Introduction

Retinoic acids, including vitamin A and its analogues, regulate the growth and differentiation. Retinoic acids suppress tumor formation in animals and have shown to be effective chemotherapeutic agents in many cancers. Predominantly all-trans retinoic acid (ATRA) and its stereoisomer 9-cis retinoic acid were found to be very potent metabolites of retinol exerting pleiotropic effects on many different biological processes. In contrast to all-trans and 13-cis, 9-cis retinoic acid has a greater effect on cell morphology. It has been reported that 13-cis retinoic acid decreases the development of primary tumors in patients with head and neck cancer, whereas ATRA provides a specific therapy for acute promyelocytic leukemia [1, 2]. The antitumor effect of retinoids is most often attributed to the induction of differentiation, but these compounds were also shown to stop the growth of tumor cells by inducing apoptosis or accelerated senescence [3]. The ability of retinoic acid to induce differentiation in cancer cells suggests their potential role as a cancer chemotherapeutic agent.

Retinoids exert their effects by modulation of gene expression by two distinct classes of nuclear receptors: retinoic acid receptors (RAR *α*, *β*, *γ*) and rexinoid receptors (RXR *α*, *β*, *γ*). The receptors belong to the steroids or thyroid hormone super-family [4]. These receptors bear six domains: A/B for ligand-independent transactivation, C with two zinc fingers for DNA binding, D is a hinge region and E is responsible for dimerization with RXR, ligand binding, ligand-dependent transactivation, and association with corepressor (CoR) or coactivators (CoA) complex. The function of F is still not known [5]. Upon ligand binding, receptors bind to retinoic acid response elements (RAREs), specific DNA sequence, as receptor dimer. ATRA binds with high affinity (kd 0.2–0.4 nM) to RARs, but not to RXRs, whereas 9-cis retinoic acid binds to RXR with high affinity [4]. RARs may exist as homodimer and can form heterodimer with RXRs as well and interact with RARE [6, 7]. The receptor dimer complex remains silenced by the binding of corepressors. Retinoid ligand binding to the receptor dimer complex induces a conformational change that removes the corepressors and recruits coactivators, thus, facilitating initiation of transcription [8]. Two types of cellular retinoic acid binding protein CRABP I and II can act as transporter of retinoic acid to facilitate the transport of cytoplasmic retinoic acid into the nucleus leading to ligand-receptor interaction [4]. 

For tumor invasion and migration, the tumor cells and host coordinately regulate matrix degradation, cell-cell attachments, and cell matrix attachment. Aberrant expression and activity of MMPs are associated with malignant development of tumor cells [9]. Most of the MMPs are secreted from cells in their inactive pro forms. The activation mechanism usually involves cleavage of the prodomain in the amino side of the conserved cystein residue followed by opening of the active site through the breakage of zinc-cystein bond [10]. The gelatinases are distinguished by the presence of an FN-like region, inserted in the catalytic domain. This insertion is important in substrate recognition [11]. Among MMPs gelatinases demonstrate specific cell-MMP interaction. For example, MMP-2 binds to integrin *α*_v_ *β*_3_ and MMP-9 to CD44 [12]. Expression of integrin *α*_v_ *β*_3_ on the cell surface supports maturation and activation of MMP-2 [13]. The membrane bound MMPs (MT-MMPs) are characterized by a c-terminal transmembrane portion and a short furin-like sequence between the pro and the catalytic domain. The furin site provides an alternative cleavage site for MMP activation [14].

MMP activity can induce tumor growth or survival, invasion, angiogenesis, and cell migration [15]. The expression of MMPs in tumor cells is regulated in a paracrine manner. Tumor cells produce factors, such as chemokines, cytokines, and the extra-cellular matrix metalloproteinase inducer (EMMPRIN), which in turn upregulate MMPs [16]. MMPs are the main group of proteolytic enzymes that facilitate tumor cell migration by degrading the basement membrane and other components of extra-cellular matrix (ECM) [17]. Beside degradation of ECM, tumor cell invasion and migration rely on the receptor-dependent attachment and release from the matrix and other cells. MMP activity directly modulates the cell-cell and cell-matrix attachment and regulates the process of cell invasion and migration [18]. Integrins play central role in anchorage-dependent growth, apoptosis, differentiation, and migration. Integrins differentially regulate the production of MMPs. In human melanoma cell lines, *α*5*β*1 integrin and *α*_v_ *β*_3_ integrin modulates release of MMP-2 and subsequent invasive behaviour [19]. Reduction of cell adhesion is of major importance in tumor metastasis and can be achieved by a variety of mechanisms affecting the E-cadherin-catenin complex. These include reduction or loss of E-cadherin expression, mutation of the genes of constituent molecules, redistribution of E-cadherin within the cell and shedding of E-cadherin, and so forth, [20].

In this communication, we report multifunctional effect of ATRA on (i) gelatinase-A expression and activity, (ii) various signaling molecules, and (iii) the integrin group of cell surface receptors in human breast cancer cells, MCF-7.

## 2. Materials

Minimal Essential Medium (MEM), Dulbecco's Modified Egal's Medium (DMEM), and foetal bovine serum (FBS) were purchased from Invitrogen Corporation, USA. Fibronectin (440 kDa) and protein G agarose were purchased from Roche, Germany. Gelatin Sepharose 4B beads was purchased from Amersham Biosciences, USA. All-trans Retinoic Acid (ATRA) was purchased from Sigma. Anti-FAK, anti-TIMP-2, anti-NF-*κ*B (p65), anti-EMMPRIN, anti-MT1-MMP, anti-E-cadherin, anti-RAR, anti-CRABP, anti-ERK, anti-phospho-ERK, and anti-VEGF antibodies were purchased from Santa Cruz, USA. Alkaline phosphatase coupled secondary antibodies (both monoclonal and polyclonal), HRP-coupled secondary antibody were purchased from Calbiochem and Nitro blue tetrazolium/5-bromo-4-chloro-3-indoyl phosphate (NBT/BCIP, western blue stabilized substrate for alkaline phosphatase), and Tetramethyl benzidine (TMB) was purchased from Bangalore Genei, India. RNAqueous 4 PCR (Total RNA isolation kit) and Retroscript (RT-PCR Kit) were purchased from Ambion, USA. MMP-2, FAK, *α*5, *β*1 primers were purchased from Operon, USA. GAPDH primers and 100 base pair DNA ladder were from Genie, Bangalore.

## 3. Methods

### 3.1. Cell Culture

A375 human melanoma cell line and MCF-7 human breast cancer cell line was obtained from National Centre for Cell Sciences (NCCS), Pune, India. MCF-7 cells were grown and maintained in MEM containing 10% FBS and A375 cells were grown in DMEM supplemented with 10% FBS in a CO_2_ incubator at 37°C.

### 3.2. Drug Treatment

7.5 mg Retinoic acid (purchased from Sigma, USA) was dissolved in 5 mL DMSO to prepare 5 mM stock solution. ATRA was added to the experimental dishes at concentrations of 10, 20, 30 *μ*M.

### 3.3. Cell Viability Assay

A375 cells (300,000/mL) and MCF-7 cells (300,000/1.5 mL) were grown in serum free culture medium (SFCM) in absence (Control) and in presence of 30 *μ*M ATRA for 24 (in case of MCF-7) and 48 (in case of A375) hours. Control and ATRA-treated cells were collected by trypsinization. 10 *μ*L of cell suspension (Control and ATRA-treated) in PBS were taken and 10 *μ*L Trypan blue was added into it. Cell suspensions were mixed well and kept for 3–5 minutes. A number of stained and unstained cells were counted in a haemocytometer slide.

### 3.4. Zymography

MCF-7 cells (300,000/1.5 mL) were initially grown in MEM supplemented with 10% FBS in petridishes, washed with serum-free culture medium (SFCM), and treated with increasing concentrations of ATRA (10 *μ*M, 20 *μ*M, 30 *μ*M) for 24 hours in SFCM. Control cells were grown without ATRA but in presence of 1% DMSO (solvent for ATRA) for 24 hours and SFCM were collected. The matrix metalloproteinases were separated from SFCM using Gelatin Sepharose 4B beads and shaking for 2hours at 4°C. The beads were washed x3 with Tris-buffered saline with Tween-20 (TBS-T) and suspended in 50 *μ*L of 1X sample buffer (0.075 gm Tris, 0.2 gm SDS in 10 mL water, pH 6.8). The suspension was incubated for 30 minutes at 37°C and then centrifuged at 3000 r.p.m. for 3 minutes. The supernatant was then subjected to zymography on 7.5% SDS-PAGE copolymerized with 0.1% gelatin. Gel was washed in 2.5% Triton-X-100 for 30 minutes to remove SDS and was then incubated overnight in reaction buffer (50 mM Tris-HCl pH 7, 4.5 mM CaCl_2_, 0.2 M NaCl). After incubation, the gel was stained with 0.5% Coomassie blue in 30% methanol and 10% glacial acetic acid. The bands were visualized by destaining the gel with water.

### 3.5. Enzyme Linked Immunosorbent Assay (ELISA)

MCF-7 cells (300,000/1.5 mL) were grown in serum-free culture medium (SFCM) in absence (Control) and in presence of 30 *μ*M ATRA (Experimental) for 24 hours. The culture supernatants were collected by centrifugation at 3000 r.p.m for 3 minutes. The wells of microtitre plate were coated in triplicate with 50 *μ*L culture SFCM and with 50 *μ*g protein from both control and experimental set and kept at 4°C overnight (plate was wrapped in wrap to prevent evaporation). Blank wells (with buffer in which samples are suspended) were also prepared. Next day wells were washed with blocking buffer (1% BSA in PBS) to block nonspecific binding sites and incubated for 1 hour at 37°C. Then the wells were washed thrice with Washing Buffer (0.5% NP-40 & 0.5% BSA dissolved in PBS). Anti-TIMP-2, anti-E-cadherin, anti-RAR, and anti-CRABP primary antibody solution (1 : 1000 dilution) was added to the wells and incubated at 37°C for 1 hour. Wells were washed thrice with Washing Buffer. Respective second antibody solution (1 : 1000 dilution buffer) was added to wells and incubated at 37°C for 1 hour. Wells were washed six times with Washing Buffer (3–5 minutes per wash). Substrate (TMB) was added to the wells (in darkness) and kept as long as required (i.e., until color developed begins to become too intense). Then 1 M H_2_SO_4_ stop solution was added and reading was taken in ELISA reader at 450 nm.

### 3.6. RT-PCR

RNA was extracted from 1 × 10^6^ MCF-7 cells grown in absence (Control) presence and of 30 *μ*M ATRA for 24 hours. The sequence of the primers used for PCR was: hMMP-2: 5′-GTA TTT GAT GGC ATC GCT CA-3′ (forward) and 5′-CAT TCC CTG CAA AGA ACA CA-3′ (reverse), hFAK: 5′-GCG CTG GCT GGA AAA AGA A-3′ (forward) and 5′-TCG GTG GGT GCT GGC TGG TAG G-3′ (reverse), h*α*5: 5′-CAT TTC CGA GTC TGG GCC AA-3′ (forward) and 5′-CAA AAC AGC CAG TAG CAA CAA-3′ (reverse), h*β*1 : 5′-TGT TCA GTG CAG AGC CTT CA-3′ (forward), and 5′-CCT CAT ACT TCG GAT TGA CC-3′ (reverse). GAPDH primers 5′-CGG AGT CAA CGG ATT TGG TCG TAT-3′ (forward) and 5′-AGC CTT CTC CAT GGT GGT GAA GAC-3′ (reverse) were used as control to normalize for mRNA integrity and equal loading. RT-PCR was carried out using two steps RT-PCR kit (Ambion, USA). Components were incubated at 42°C for 1 hour and at 92°C for 10 minutes (to inactivate the reverse transcriptase) Conditions used for PCR consisted of 24 cycles for MMP-2 at 94°C for 30 seconds, 63°C for 30 seconds, and 72°C for 60 seconds, 25 cycles for FAK at 94°C for 30 seconds, 60°C for 30 seconds and 72°C for 1 : 30 minutes and 28 cycles for *α*5 & *β*1 at 94°C for 30 seconds, 58°C for 30 seconds, and 72°C for 1 : 30 minutes with a final incubation at 72°C for 7 minutes in DNA thermal cycler. The predicted size of the PCR products were 198 base pairs (bp) for MMP-2, 476 bps for FAK, 324 for *α*5, 452 for *β*1, and 454 for GAPDH.

### 3.7. Immunoblot Assay of MT1-MMP, EMMPRIN, FAK and NF-*κ*B

MCF-7 cells (300,000/1.5 mL) were grown in serum free culture medium (SFCM) in absence (control) and in presence of 30 *μ*M ATRA for 24 hours. The respective cells were collected. Cell extraction was carried out using cell extraction buffer (37.5 mM Tris, 75 mM NaCl and 0.5% Triton-X-100) and the protein content of the extracts were estimated by Lowry's method. Equal amount of protein was taken and incubated with 1X sample buffer for 30 minutes followed by 5–8 minutes incubation with 0.1 volumes *β*-mercaptoethanol at 80–90°C. Samples were then subjected to electrophoresis on 7.5% SDS-PAGE. The proteins were transferred on to nitrocellulose membranes by Western Blot at 300 mA for 3 hours. The membranes were blocked with 1% BSA and subsequently washed thrice with TBS-T. The immunoblots were reacted with anti-MT1-MMP, anti-EMMPRIN, anti FAK, anti-NF-*κ*B and anti-Ig-G antibodies, respectively, (1 : 1000 dilution) for 1.5 hours at 37°C followed by incubation with respective alkaline phosphatase coupled second antibodies. Bands were developed using NBT-BCIP as substrate.

### 3.8. Immunoblot Assay of ERK, Phospho-ERK and VEGF by Immunoprecipitation

MCF-7 cells (300,000/1.5 mL) were grown in serum-free culture medium (SFCM) in absence (Control) and in presence of 30 *μ*M ATRA for 24 hours. The cells were collected. Cell extraction was carried out using the cell extraction buffer and the protein content of the extracts was estimated by Lowry's method. Equal amount of protein was taken from each extract and ERK, VEGF was immunoprecipitated from the extracts using anti-ERK, anti-VEGF, and anti-Ig-G antibodies and protein-G agarose beads and shaking overnight at 4°C. The resultant immune-complex was washed thrice in PBS, suspended in 1X sample buffer and incubated at 37°C for 30 minutes, followed by incubation with 0.1 volumes of *β*-mercaptoethanol for 5–8 minutes at 80–90°C. Samples were then subjected to electrophorese on 7.5% SDS-PAGE. The proteins were transferred on to nitrocellulose membrane by Western Blot. The membranes were blocked with 1% BSA and subsequently washed thrice with TBS-T. The immunoblots were reacted with anti-ERK antibody, anti-phospho-ERK antibody, anti-VEGF, and anti-Ig-G antibodies, respectively, in 1 : 1000 dilution, and kept at 37°C for 1.5 hours. After washing with TBS-T, membranes were incubated with alkaline phosphatase coupled respective second antibodies (1 : 1000 dilutions). Bands were visualized using NBT/BCIP as substrate.

### 3.9. Cell Adhesion Assay

The microtitre plate wells were coated with fibronectin (1.56 *μ*g, 3.125 *μ*g, 6.25 *μ*g, 12.5 *μ*g, and 25 *μ*g fibronectin/well). The ligands were allowed to bind for 1.5 hours at 37°C. Wells were blocked with Buffer C (1% BSA, 1 mM CaCl_2_, and 1 mM MgCl_2_ in PBS) for 1 hour at 37°C. Control and 30 *μ*M ATRA-treated (for 24 hours in complete medium) MCF-7 cells were trypsinised from culture dishes, washed, suspended in Buffer C, added to microtitre plates (50,000 cells/well), and allowed to bind at 37°C for 1.5 hours. The wells were washed ×3 with Buffer C. The bound cells were trypsinised, counted on haemocytometer, and expressed as % of adhesion**.**

### 3.10. Statistical Analysis

Statistical analyses were done with Student's *t*-test comparing between the control and treated groups.

## 4. Results

### 4.1. Effect of ATRA on Cell Viability

**Figure 1:**
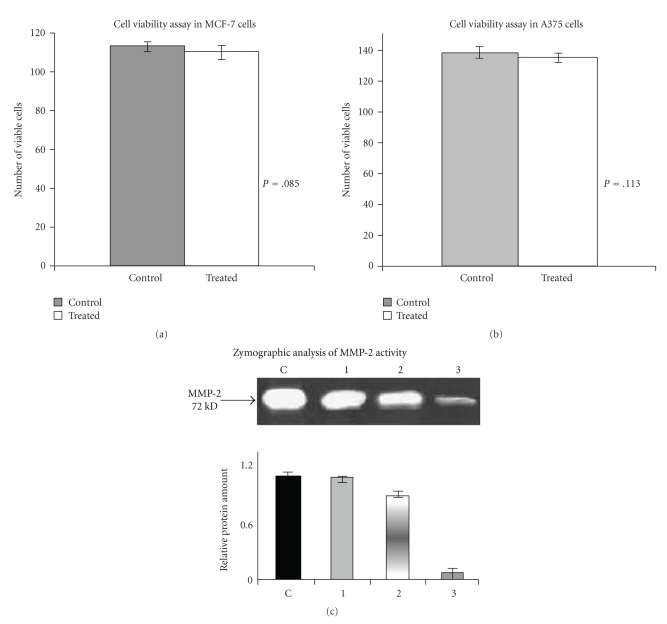
*Cell viability assay*: MCF-7 (300,000 cells/1.5 mL) (Figure 1(a)) and A375 (300,000/mL) (Figure 1(b)) cells were grown in serum-free culture medium (SFCM) in absence (Control) and in presence of 30 *μ*M ATRA (Treated) for 24 hours (Figure 1(a)) and 48 hours (Figure 1(b)). Cells were collected and 0.4% trypan blue solution was added in both control and ATRA-treated cell suspension. Number of blue cells and nonblue cells were counted and *t*-test was done to obtain the *P*-values. *Zymographic analysis of pro-MMP-2 activity*: MCF-7 (300,000 cells/1.5 mL) cells were grown in serum-free culture medium (SFCM) in absence (control) (lane C) and in presence of 10 (lane 1), 20 (lane 2), 30 (lane 3) *μ*M ATRA for 24 hours (Figure 1(c)). The culture supernatants were collected and MMPs were concentrated using Gelatin-sepharose 4B beads. Gelatinases were eluted from bead with sample buffer and subjected to zymography on 7.5% SDS-PAGE copolymerised with 0.1% gelatin. The zymogram was treated with 2.5% triton X-100 for 30 minutes followed by incubation in reaction buffer for 20 hours and stained with coomassie blue. The quantitative measurement of the zymogram (Figure 1(c)) was performed by using Image J Launcher (version 1.4.3.67). (C) shows the pro-MMP-2 activity in the SFCM of control cells. (1) is the representative of pro-MMP-2 activity in the SFCM of 10 *μ*M ATRA-treated cells. (2) and (3) represents the pro-MMP-2 activity in the SFCM of 20 and 30 *μ*M ATRA-treated cells (for 24 hours), respectively.

Cell viability in MCF-7 (Figure 1(a)) cells before and after ATRA treatment was checked using trypan blue stain (0.4%). In case of control 113 cells (average of 3 experiments) in 0.1 *μ*L (Control) were counted as viable and in treated average of 3 experiments showed 110 cells/0.1 *μ*L (Treated) were viable. However, *T*-test showed that there was no statistically significant difference (*P* = .085) between two groups. Therefore, no changes were observed in cell viability of control and ATRA-treated.

Figure 1(b) showed the cell viability in A375 cells. In control, 138 cells/0.1 *μ*L (average of 3 experiments) and in treated 134 cells/0.1 *μ*L (average of 3 experiments) were viable. *P* = .113 indicates that there is no statistically significant difference in the cell viability of ATRA untreated (Control) and ATRA-treated (Treated) cells.

### 4.2. Effect of ATRA on Pro-MMP-2 Activity

The comparative zymogram (Figure 1(c)) of control (lane C) MCF-7 cells and MCF-7 cells treated with 10 *μ*M (lane 1), 20 *μ*M (lane 2) and 30 *μ*M (lane 3) ATRA for 24 hours showed appreciable reduction in pro-MMP-2 activity in the SFCM of 30 *μ*M ATRA-treated MCF-7 cells as compared to that of the control. Pro-MMP-2 activity was not appreciably inhibited after 10 *μ*M and 20 *μ*M ATRA treatment for 24 hours. Pro-MMP-2 activity of control and 30 *μ*M ATRA-treated (for 12 hours) MCF-7 cells in SFCM showed no appreciable change in gelatinolytic activity.

### 4.3. Effect of ATRA on TIMP-2 Protein Expression

**Figure 2:**
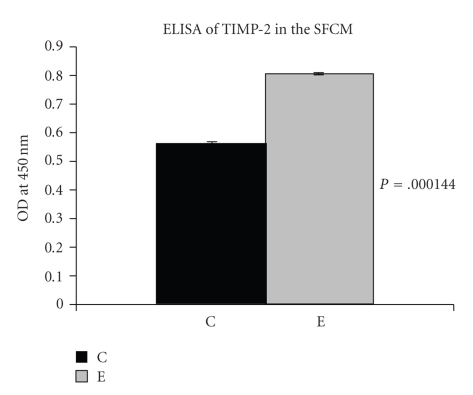
*ELISA of TIMP-2, E-cadherin, RAR* and *CRABP*: MCF-7 (300,000 cells/1.5 mL) cells were grown in serum free culture medium (SFCM) in absence (C) and in presence of 30 *μ*M ATRA (E) for 24 hours. The culture supernatants were collected and the wells of an ELISA plate was coated with 50 *μ*L SFCM (for TIMP-2) and 50 *μ*g of protein (for E-cadherin, RAR & CRABP) from both control and ATRA-treated cells and kept at 4°C for overnight. The next day contents of the wells were discarded and the wells were washed with blocking buffer. Then 50 *μ*L of blocking buffer was added to each well and kept at 37°C for 1 hour. The blocking buffer was discarded and the wells were washed with washing buffer. The wells were then reacted with anti-TIMP-2 (Figure 2), anti-Ecadherin (Figure 5(c)), anti-RAR (Figure 8(a)), anti CRABP (Figure 8(b)) antibody, respectively, and kept in 37°C for 1 hour. The wells were washed with washing buffer and reacted with respective HRP-coupled secondary antibody and kept in 37°C for 1 hour. The wells were washed with washing buffer and then TMB substrate was added to each well for color development. The color reaction was stopped with 1 M H_2_SO_4_ solution and OD of each well was measured at 450 nm. The OD indicated the expression level of TIMP-2 (*P* = .000144 (*P* < .05)) in control and treated MCF-7 cells.

When the culture supernatant from both control and 30 *μ*M ATRA-treated MCF-7 cells were assayed for MMP-2 and tissue inhibitor of MMP-2 (TIMP-2) protein by ELISA (Figure 2), it was found that ATRA substantially increased TIMP-2 protein level. *P* = .000144 (*P* < .05) indicates data obtained are highly significant.

### 4.4. Effect of ATRA on MT1-MMP Expression

**Figure 3:**
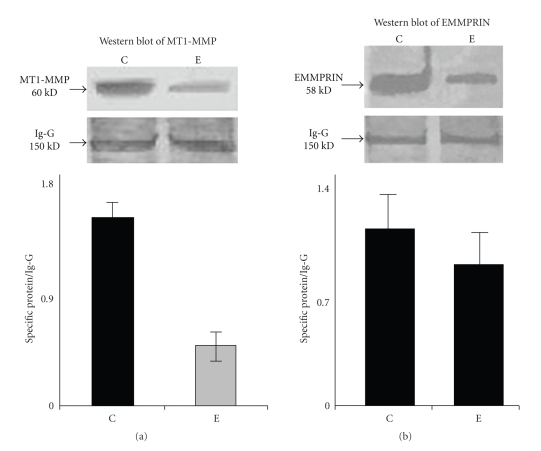
*Western blot of MT1-MMP and EMMPRIN*: MCF-7 (300,000 cells/1.5 mL) cells were grown in absence (lane C) and presence of 30 *μ*M ATRA (lane E) for 24 hours in SFCM. The cells were collected and were extracted in cell extraction buffer. 50 *μ*g of protein from both control and ATRA-treated cell extracts were run on 7.5% SDS-PAGE and the proteins were transferred onto nitrocellulose membrane by western blot. The membrane was incubated with anti MT1-MMP (Figure 3(a)) and EMMPRIN (Figure 3(b)) antibody and then after washing membrane was incubated with respective alkaline phosphatase coupled secondary antibody. Bands were visualized using NBT-BCIP as substrate. Ig-G was used as internal control. The quantitative measurement of the western blot (Figures 3(a) and 3(b)) was performed by Image J Launcher (version 1.4.3.67). (C) is the amount of MT1-MMP and EMMPRIN expression in the control cells, respectively, and (E) is the amount of MT1-MMP and EMMPRIN expression of 30 *μ*M ATRA-treated (for 24 hours) MCF-7 cells, respectively.

To study the status of pro-MMP-2 activation complex, we studied the membrane type 1 MMP (MT1-MMP) expression by western blot using anti-MT1-MMP polyclonal antibody. The result (Figure 3(a)) showed that 30 *μ*M ATRA-treated (lane E) MCF-7 cells exhibit much lower expression of MT1-MMP compared to the untreated MCF-7 cells (lane C).

### 4.5. Effect of ATRA on EMMPRIN Expression

Treatment with 30 *μ*M ATRA for 24 hours (lane E) in MCF-7 cells showed appreciable downregulation in EMMPRIN expression compared to the control (lane C) as analyzed by western blot (Figure 3(b)).

### 4.6. Effect of ATRA on MMP-2 Gene Expression

**Figure 4:**
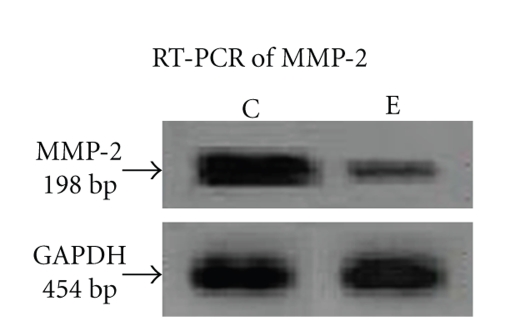
*RT-PCR of MMP-2*: MCF-7 (300,000 cells/1.5 mL) cells were grown in absence (lane C) and in presence of 30 *μ*M ATRA (lane E) for 24 hours in SFCM. Cells were washed in PBS and total RNA were extracted (RNAqueous for PCR, Ambion). Two-step RT-PCR (Retroscript, Ambion) was done with equal amounts of total RNA using specific primers for MMP-2 PCR. 20 *μ*L of each PCR products were run on a 2.5% agarose gel and bands visualised under UV. GAPDH primers were used to confirm equal loading. Documentation was done in Gel Doc (Image Master VDS, Pharmacia, Biotech).

To analyze whether ATRA affects MMP-2 by regulating at transcription levels, we studied mRNA expression for MMP-2 by RT-PCR. Figure 4 demonstrates a reduction in MMP-2 mRNA expression after treating MCF-7 cells with 30 *μ*M ATRA for 24 hours (lane E) comparing the control (lane C).

### 4.7. Effect of ATRA on Integrin Receptor—Ligand Interaction

**Figure 5:**
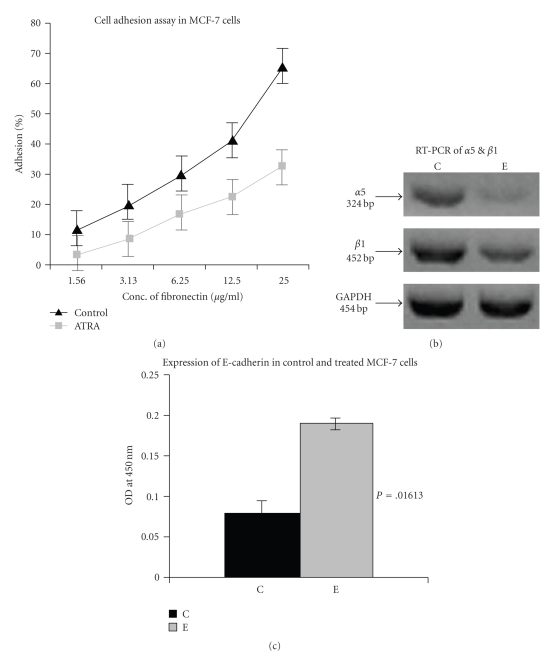
(a) *Cell Adhesion Assay*: MCF-7 cells (300,000 cells/1.5 mL) were grown in absence (Control) and in presence of 30 *μ*M ATRA for 24 hours in 10% MEM. Microtitre wells were coated with various concentrations of fibronectin and kept at 37°C for 1.5 hour. 1% BSA solution was used to block the nonspecific sites. Control and ATRA-treated cells were collected by trypsinization and 50,000 cells were added to each well. After 1 hour of incubation wells were washed thoroughly, number of bound cells was counted on a haemocytometer, and the % of cells adhered to the ligand was calculated. (b) *RT-PCR of* *α*5 & *β*1: MCF-7 (300,000 cells/5 mL) cells were grown in absence (lane C) and in presence of 30 *μ*M ATRA (lane E) for 24 hours in SFCM. Two steps RT-PCR (Retroscript, Ambion) was done as before with equal amounts of total RNA using specific primers for *α*5 & *β*1. GAPDH was used as internal control. (c) *ELISA of E-cadherin*: ELISA of E-cadherin was performed with anti-E-cadherin antibody in control (C) and ATRA treated (E) MCF-7 cells. *P* = .01613 (*P* < .05) indicates that the difference in E-cadherin expression between control and ATRA treated MCF-7 cell is statistically significant.

Figure 5(a) shows the effect of 30 *μ*M ATRA for 24 hours on adhesion of MCF-7 cells to ECM protein (fibronectin). Binding of cells to fibronectin decreased significantly upon ATRA treatment for 24 hours whereas binding of cells to fibronectin remained almost unaltered for 12 hours ATRA-treated cells (result not shown).

### 4.8. Effect of ATRA on *α*5 and *β*1 Integrin Receptor Expression

RT-PCR analysis (Figure 5(b)) of control and ATRA-treated cells showed significant down regulation of *α*5 expression in ATRA-treated cells. *β*1 expression was also downregulated upon 30 *μ*M ATRA treatment for 24 hours.

### 4.9. Effect of ATRA on E-Cadherin Expression

Figure 5(c) represents the status of E-cadherin in control (C) and 30 *μ*M ATRA-treated (E) MCF-7 cells. When the expression level of E-cadherin protein in control and 30 *μ*M ATRA-treated (for 24 hours) were measured by ELISA, result showed that E-cadherin expression increased significantly upon ATRA treatment. *P* = .01613 (*P* < .05) indicates statistically significant difference in E-cadherin expression between control and ATRA-treated cells.

### 4.10. Effect of ATRA on FAK Expression

**Figure 6:**
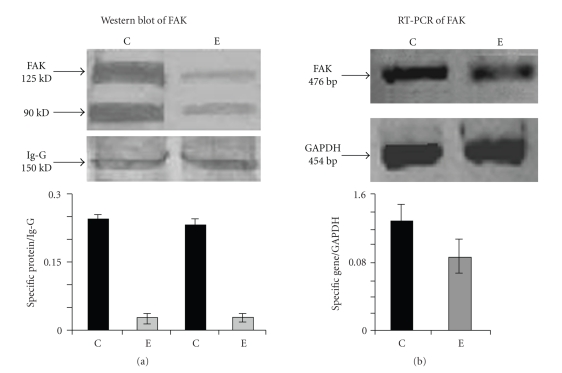
(a) *Western blot analysis of FAK*: MCF-7 (300,000 cells/1.5 mL) cells were grown in absence (lane C) and presence of 30 *μ*M ATRA (lane E) for 24 hours in SFCM. The cells were collected and were extracted in cell extraction buffer. 50 *μ*g of protein from both control and ATRA-treated cell extracts were run on 7.5% SDS-PAGE and the proteins were transferred onto nitrocellulose membrane by western blot. The membrane was incubated with anti-FAK antibody followed by incubation with alkaline phosphatase coupled secondary antibody. Bands were visualized using NBT-BCIP as substrate. Quantitative measurements of immunoblot was done by using Image J Launcher (version 1.4.3.67). (C) represents the expression of respective proteins in control cells whereas (E) represents expression in 30 *μ*M ATRA treated cells. (b) *RT-PCR of FAK:* Figure 6(b) showed the status of FAK mRNA expression in control (lane C) and ATRA treated (lane E) MCF-7 cells. A quantitative measurement of RT-PCR (Figure 6(b)) was done by using Image J Launcher (version 1.4.3.67). (C) represents the expression of FAK in control cells whereas (E) represents FAK expression 30 *μ*M ATRA treated cells in respective figures.

Focal adhesion kinase (FAK) is located at the upstream of signaling cascade, especially the integrin signaling pathway involves FAK. In order to determine if ATRA affect the FAK pathway in MCF-7 cells, cells were cultured in absence (lane C) and presence of 30 *μ*M ATRA (lane E) for 24 hours and FAK status was then determined by RT-PCR and western blot analysis. Result showed that FAK (Figures 6(a)  and 6(b)) expression was reduced upon ATRA treatment.

### 4.11. Effect of ATRA on NF-kB Expression

Nuclear factor kB (NF-kB) is one of the important signaling molecules affecting the activation of pro-MMP-2. Therefore, the status of NF-kB in control (lane C) and 30 *μ*M ATRA (lane E) treated MCF-7 cells was assayed by western blot. Figre 7(a)  is the representative of the western blot of NF-kB showed that the expression of NF-kB protein was appreciably reduced upon 30 *μ*M ATRA treatment for 24 hours in MCF-7 cells.

### 4.12. Effect of ATRA on ERK and p-ERK

**Figure 7:**
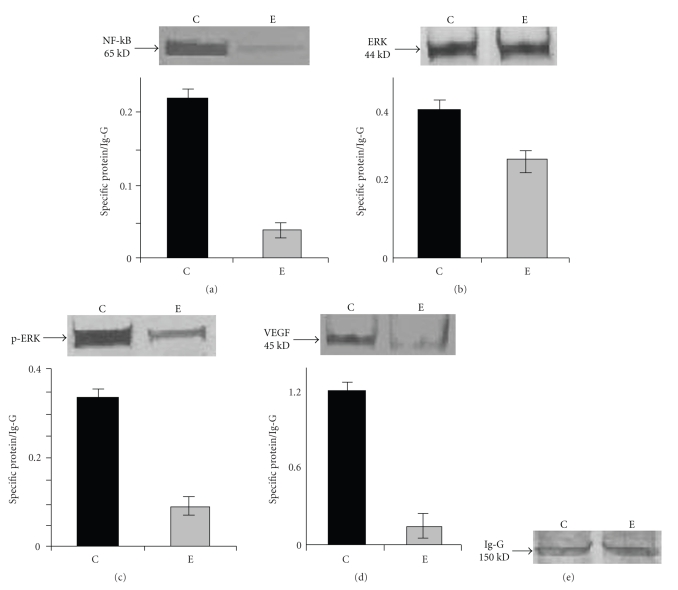
(a) *Western blot of NF-kB*: NF-kB expression in control (lane C) and ATRA treated (lane E) MCF-7 cells was observed by western blot analysis as before using anti-NF-*κ*B primary antibody. Quantitative measurements of immunoblots were done by using Image J Launcher (version 1.4.3.67). (C) represents the expression of respective proteins in control cells whereas (E) represents expression in 30 *μ*M ATRA treated cells. (b), (c), and (d) *Immunoblot assay of ERK, p-ERK, and VEGF: * MCF-7 cells (300,000 cells/1.5 mL) were grown in absence (lane C) and presence of 30 *μ*M ATRA (lane E) for 24 hours in SFCM. Cells were collected and extracted in cell extraction buffer. ERK and PI-3K were immunoprecipitated from 150 *μ*g of protein from both control and ATRA-treated cell extract with anti-ERK & protein G Agarose bead (Figures 7(b) and 7(c)) and with anti-VEGF & protein G Agarose bead (Figure 7(d)), keeping the samples for overnight at 4°C with shaking. In each case the resultant immune complex was washed thrice with PBS and the respective protein bound with antibody were eluted from the agarose bead using 1X sample buffer. Samples were then incubated in *β*-mercaptoethanol for 10 minutes at 90°C. Samples were subjected to electrophoresis on 7.5% SDS-PAGE. The proteins were transferred to nitrocellulose membrane by Western Blot. The membranes were incubated with anti-ERK (Figure 7(b)), anti-phospho ERK (Figure 7(c)), and anti-VEGF antibody (Figure 7(d)), respectively. The immunoblots were then incubated with alkaline phosphatase-coupled secondary antibodies and bands were visualized by NBT/BCIP substrate. Ig-G (Figure 7(e)) was used to confirm equal loading. Quantitative measurements of immunoblots were done by using Image J Launcher (version 1.4.3.67). (C) represents the expression of respective proteins in control cells whereas (E) represents expression in 30 *μ*M ATRA treated cells.

Figures 7(b)  and 7(c)  show the comparative ERK (Figure 7(b)) and p-ERK (Figure 7(c)) expression by immunoprecipitation of ERK and p-ERK from whole cell extract with anti ERK and anti p-ERK antibodies followed by western blot. The results show that ATRA treatment downregulates the phosphorylation of ERK, but the ERK expression was not found to be altered significantly after ATRA treatment. The expression of p-ERK is much less in the ATRA-treated (lane E) MCF-7 cell extract compared with untreated (lane C) MCF-7 cell extract.

### 4.13. Effect of ATRA on VEGF Expression

When the expression of VEGF protein was assayed in ATRA-treated (lane E) and untreated (lane C) MCF-7 cells, western blot (Figure 7(d)) demonstrated appreciable reduction in VEGF protein expression in 30 *μ*M ATRA-treated MCF-7 cells, compared to that of the control cells.

### 4.14. Effect of ATRA on RAR and CRABP Expression

**Figure 8:**
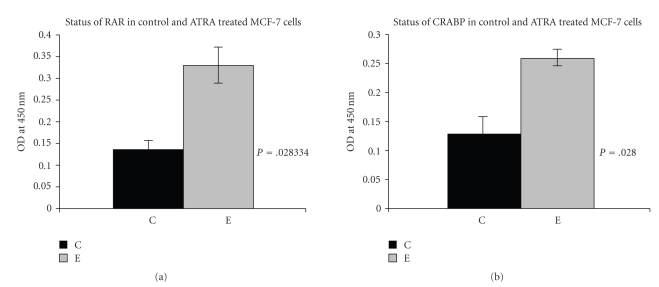
*ELISA of RAR* & *CRABP*: The status of RAR (Figure 8(a)) and CRABP (Figure 8(b)) in control (C) and ATRA treated (E) MCF-7 cells were analysed by ELISA as before using anti-RAR and anti-CRABP primary antibody and respective secondary antibodies. The OD indicates the expression level of RAR (Figure 8(a); *P* = .028334 (*P* < .05)) and CRABP (Figure 8(b); *P* = .028 (*P* < .05)) in whole cell extract.

ELISA was performed to observe the status of RAR (Figure 8(a)) and CRABP (Figure 8(b)) upon ATRA treatment and it was found that RAR and CRABP expression increased appreciably in 30 *μ*M ATRA-treated (E) MCF-7 cells, compared to that of the control cells(C). *P* = .028 (*P* < .05) indicates that differences between control treated cells are statistically significant.

## 5. Discussion

In this communication we report the effect of all-trans retinoic acid (ATRA) on the expression and activity of matrix metalloproteinases and possible mechanism that may influence the drug to exert its effect. Treatment of MCF-7 cells with ATRA showed appreciable inhibition of pro-MMP-2 enzyme expression and activity. Treating cells with 30 *μ*M ATRA showed significant induction of the TIMP-2 protein expression in the SFCM. The activity of MMPs in extra cellular space is specifically inhibited by tissue inhibitors of metalloproteinases (TIMPs). TIMPs bind to the highly conserved zinc binding site of active MMPs at molar equivalence. Over expression of TIMP-2 can inhibit the activity of MMP-2 [21] and it also inhibits the invasive and metastatic behaviors of cancer cells. Braunhut et al. showed that retinoic acid upregulated TIMP-2 expression in endothelial cells [22]. Other studies also showed a pronounced induction of the TIMP-1 protein by vitamin A [23, 24]. RT-PCR result demonstrates appreciable reduction of MMP-2 gene expression upon 30 *μ*M ATRA treatment for 24 hours. Therefore, ATRA could suppress the gelatinolytic enzyme activity by regulating MMP-2 gene transcription. Pro-MMP-2 is activated at the cell surface through a unique multistep pathway that involves *α*_v_ *β*_3_ integrin, MT1-MMP and TIMP-2. MT1-MMP acts as receptor and activator for pro MMP-2. However, MT1-MMP cannot bind pro MMP-2 directly, and TIMP-2 acts as an adaptor molecule to mediate pro MMP-2 binding by forming ternary complex. The N-terminal domain of TIMP-2 binds the hemopexin like domain of pro MMP-2. Cleavage of pro MMP-2 requires the presence of free MT1-MMP in addition to the complex [25, 26]. Our results demonstrate ATRA mediated reduction of MT1-MMP protein expression which may cause reduced activation of pro-MMP-2 in ATRA-treated MCF-7 cells. Extracellular matrix metalloproteinase inducer (EMMPRIN), a 58 kD type-I trans membrane protein is known to play role in ECM remodeling through the activation of MMP production [27]. EMMPRIN exhibits potential to induce MMP-2 expression [28]. In this study downregulation of EMMPRIN expression by ATRA may cause reduced pro-MMP-2 activity in the SFCM of ATRA-treated MCF-7 cells.

The interaction of cells with adhesion protein in the ECM provides signals which affect the morphology, motility, gene expression and survival of adherent cells [29]. The relationship that exists between integrin receptor, fibronectin, and MMP expression is of particular interest. Fibronectin is a prototype cell adhesion protein. It is present as a polymeric fibrillar network in the ECM and as soluble protomer in body fluids. Two regions in each fibronectin subunit possess cell binding activity: III_9-10_ and III_14-V_. The RGD motif in fibronectin is located in III_10_ and is the most important recognition site for most of the known integrins [30]. Integrins, a family of cell surface receptors, mediate attachment of the cells to ECM and initiate a series of signaling events that ultimately activates proteases like gelatinases. Integrins mediate both cell-cell and cell-substratum adhesion and signaling [29, 31]. Binding of integrin receptors to fibronectin can initiate organization of the cytoskeleton and focal contacts and signals which affect gene expression. Fibronectin through interaction with its corresponding *α*_5_ *β*_1_ integrin receptor regulate the activity and expression of MMP-2 and MMP-9 [32–34]. On binding to ECM molecules, integrins are thought to undergo a conformational change, which allows the intracellular domain of their *β* subunit to interact with focal adhesion proteins. FAK appears to localize to nascent focal adhesions. Upon activation, FAK combines with Src and FAK/Src signaling pathway and promotes cell migration [35, 36]. Our study demonstrates that treatment of MCF-7 cells with ATRA downregulates the expression of *α*5 and *β*1 which reflects the result of cell adhesion assay, showing reduced tumor cell binding to ECM protein fibronectin after ATRA treatment in a time dependent manner. Our earlier study showed that ATRA treatment downregulates *α*_5_ and *β*_1_ subunit expressions of *α*_5_ *β*_1_ receptor in SiHa cells [37]. Focal adhesion kinase (FAK) was first shown to be linked to integrin signaling when it was identified as a major substrate for tyrosine phosphorylation. It has been suggested that Rho, a Ras-like GTP binding protein that regulates the formation of focal adhesions and actin stress fibers, may be involved in the activation of FAK. Thus, multiple cancer cell survival stimuli that signal through diverse pathways converge to induce the tyrosine phosphorylation of a common substrate, FAK [38]. Furthermore, FAK induces MMP-2 secretion from cell interior to its medium [39, 40]. ATRA treatment caused a significant decrease in FAK expression in MCF-7 cells. It may contribute to the cell motility and decreased gelatinase activity by regulating the secretion of MMP-2 in the SFCM. Alterations in the adhesion properties of neoplastic cells may play a pivotal role in the development and progression of the malignant phenotype in a range of tumor types. Adhesion molecules are intimately involved in the control of such processes as morphological differentiation, cellular proliferation, and invasion and colonization at distant organs [41]. E-cadherin dependent cell-cell adhesion is important for the maintenance of epithelial structural integrity and the loss of E-cadherin expression has been shown to correlate with increased invasive potential of both carcinoma cell lines and human tumor samples [42]. Recent data demonstrate that tumor-associated MMPs can modulate cell-cell adhesion by cleaving E-cadherin [43]. Evidence is emerging that there may be cross-talk between the cadherins and the integrins. Anticadherin antibodies have been shown to prevent the loss of *α*6 and *β*1 integrins in terminally differentiating keratinocytes [44]. Positive expression of E-cadherin could decrease cell adhesion to fibronectin partially through transcriptional inhibition of *α*5*β*1 integrin gene [45]. Our results showed that ATRA treatment up-regulated E-cadherin expression in MCF-7 cells which could downregulate the *α*5 and *β*1 expression and possibly attributed to inhibition of cell-matrix adhesion and therefore in turn inhibit pro-MMP-2 activity. Overexpression of E-cadherin decreased MMP-2 activity in prostate cancer cells and MT1-MMP in squamous cancer cells [46]. Therefore, overexpression of E-cadherin in ATRA-treated MCF-7 cells may downregulate pro-MMP-2 activity in the SFCM of ATRA-treated cells.

In our study, we found decreased expression of NF-*κ*B in ATRA-treated MCF7 cells. NF-*κ*B is active in nucleus and is inhibited through its sequestration in the cytoplasm by inhibitor of *κ*B (I*κ*B). I*κ*B kinase (IKK) can degrade I*κ*B and thus release NF-*κ*B from inhibition [47]. NF-*κ*B mediates MT1-MMP induction and pro-MMP-2 activation. Osteopontin, a membrane protein induces NF-*κ*B mediated pro-MMP-2 activation through I*κ*B*α*/IKK phosphorylation [48]. Therefore, by suppressing the NF-*κ*B protein expression ATRA may downregulate pro-MMP-2 activation. Furthermore, constitutive activation of NF-*κ*B may very well play an anti-apoptotic role in the breast and prostate carcinoma cells [49]. The inhibition of constitutive NF-*κ*B activation by reducing NF-*κ*B expression in ATRA-treated MCF-7 cells may therefore enhance the basal apoptotic rate in these cells. 

Dual specificity phosphatase 6 (DUSP6) dephosphorylates activated ERK and blocks the growth stimulatory signals. DUSP6 and RGS16 (regulator of G-protein signaling 16) transcriptional upregulation inhibits tumor cell proliferation and ERK phosphorylation and serves as a negative regulatory mechanism to prevent further ERK phosphorylation. Liu et al. showed RA induced upregulation of DUSP6 & RGS16 [50]. Our study demonstrates decreased p-ERK level in ATRA-treated cells. Reduced ERK phosphorylation may result due to ATRA induced transcriptional activation of DUSP6 and RGS16. Activation of ERK plays an essential role in the expression of MT1-MMP [51, 52]. Reduced activation of ERK may cause lesser expression of MT1-MMP in ATRA-treated cells.

Angiogenesis is known to be one of the most important aggravating steps in the progression of cancer. Various angiogenic factors are known to be upregulated in cancer. Some of the proangiogenic factors such as thrombin, H_2_O_2_, hepatocyte growth factors, vascular endothelial growth factor (VEGF) are known to have contribution to the activation and release of MMP-2 [53, 54]. Upon ligand (VEGF) binding to its receptor VEGFR-2 tyrosine kinase signaling cascade gets stimulated. As a result of this signaling cascade, proliferation/survival factors (bFGF), migration factors (ICAMs/VCAMs/MMPs), and vessel permeability factors (eNOs) are produced [55]. Downregulation of VEGF expression in ATRA-treated MCF-7 cells may contribute to the reduced pro-MMP-2 activity in treated cells.

All-trans retinoic acid after entering into the cell, its entry from cytoplasm to nucleus is facilitated by CRABP I/II. In the nucleus it binds with its specific receptor RAR/RXR and exerts its effect by regulating various gene transcriptions. Several studies suggested that RAR/RXR and CRABP expression got increased when cells received ATRA treatment [56, 57]. Our study also demonstrates up-regulation of RAR and CRABP expression upon ATRA treatment in MCF-7 cells.

In conclusion, the present study demonstrates the inhibitory effect of ATRA on pro-MMP-2 activity and the possible molecular mechanisms. Our experimental findings strongly indicate that ATRA induced inhibition of pro-MMP-2 activity may result due to increased expression of TIMP-2, and downregulation of FAK, MT1-MMP, EMMPRIN, and NF-kB expression. ATRA may interfere with the integrin down-stream signaling altering the expression and activity of pro-MMP-2.

## Abbreviations

ATRA: All-trans retinoic acid
MMP: Matrix metalloproteinase
TIMP: Tissue inhibitor of metalloproteinase
MT1-MMP: Membrane type-1 matrix metalloproteinase
EMMPRIN: Extra cellular matrix metalloproteinase inducer
FAK: Focal adhesion kinase
NF-kB: Nuclear factor kB
ERK: Extra cellular signal regulating kinase
p-ERK: Phospho-ERK
PI-3K: Phosphatidyl inositol 3 kinase
p-PI-3K: Phospho-PI-3K
VEGF: Vascular endothelial growth factor
RGD: Arginine-Glycine-Aspartate
RAR: Retinoic acid receptor
CRABP: Cellular retinoic acid binding protein.
